# Quality of Life of Adopted Chinese Versus Nonadopted Dutch Children with Cleft Lip and/or Palate: A Propensity Score Matched Analysis

**DOI:** 10.1177/10556656211050795

**Published:** 2021-12-06

**Authors:** Martinus M. van Veen, Bente A. van den Berge, Chantal M. Mouës-Vink

**Affiliations:** 1 Department of Plastic Surgery, 4480 Medical Center Leeuwarden, Leeuwarden, The Netherlands; 2 Department of Plastic Surgery, 10173 University Medical Center Groningen, Groningen, The Netherlands

**Keywords:** cleft lip, cleft palate, adoption, quality of life

## Abstract

**Objective:**

To examine quality of life in internationally adopted children with cleft lip and/or palate (CL/P) versus non-adopted children with CL/P.

**Design:**

Cross sectional study.

**Setting:**

Multidisciplinary cleft team of a secondary and tertiary hospital in the Netherlands.

**Methods:**

Parents of children under the age of 8 treated by the multidisciplinary cleft team of our institutions were asked to fill out a questionnaire containing demographic and clinical data and a validated parent proxy measure of cleft-specific quality of life instrument for children aged 0–8: the CleftChild-8. Adopted children were matched to non-adopted children using propensity score matching based on sex, age, type of cleft, if palatal surgery was completed and the level of education of the parent. CleftChild-8 scores were then compared between the matched samples of adopted and non-adopted children with CL/P.

**Main Outcome Measure(s):**

Differences in (sub)domain scores of the CleftChild-8.

**Results:**

Most median CleftChild-8 scores of the adopted children (n = 29) were slightly lower compared to the 29 matched non-adopted children. A significant difference was seen for the domain score ‘satisfaction with (operative) treatment’ and 3 of the 13 subdomain scores: ‘post-operative results’, ‘acceptance by siblings’ and ‘acceptance by family/friends’.

**Conclusions:**

By parent report, adopted children with CL/P experienced some areas of lower quality of life when compared to non-adopted children. Members of cleft teams should be aware of the problems associated with adoption and offer additional guidance and counseling to adopted children and their parents.

## Introduction

The number of internationally adopted children has been decreasing for years in most western countries including the Netherlands (Stichting Adoptievoorzieningen, 2017). However, the number of adopted children with congenital diagnoses, such as cleft lip and/or palate (CL/P), remains stable, and these children form a distinct treatment challenge for Dutch CL/P treatment teams.

### Surgical Care for Internationally Adopted Children

Internationally adopted children often have complex medical needs, and may have an additional history of trauma, neglect and institutionalization (Knipper et al., 2020). Neglect and institutionalization have been shown to be associated with lasting neurobiological impairments, with a strong correlation to many behavioral, emotional and cognitive problems (Julian, 2013). After adoption, adopted children face the unique challenge of assimilating into a new culture, learning a new language and growing up in a culture where they are the minority (Juffer and Van IJzendoorn, 2005). Lastly, independent from adoption-related risk, any major medical experience can potentially lead to the development of post-traumatic stress in children (Scheeringa, 2019). Surgical care providers should be aware of the distinctive situation of internationally adopted children.

### Care for Adopted Children with Cleft lip and/or Palate

CL/P is one of the most common congenital malformations seen in internationally adopted children (Werker et al., 2017; Stichting Adoptievoorzieningen, 2017) and demographics and clinical care for these children has been described by several teams.

Children are approximately 2 years old at the time of adoption (Hansson et al., 2012; Swanson et al., 2014; Werker et al., 2017; Kaye et al., 2019). In locally born children, our team usually performs some of the major cleft surgeries prior to the age of two, such as lip repair at 3 months old, and palate closure at 9 months old. Hence, internationally adopted children often present to a CL/P team later in the typical treatment timeline. Most children already undergo surgery at their country of origin (Swanson et al., 2014; Kaye et al., 2019), with one study describing 79% of children undergoing surgery, with cleft lips being repaired more frequently compared to cleft palates (Werker et al., 2017). However, revision surgeries are needed relatively often (Kaye et al., 2019), with one CL/P team even reporting revision surgeries in virtually all adopted children (Mulliken et al., 2015). Since palatal closure has usually not yet been performed, adopted children are at higher risk of velopharyngeal insufficiency (Sullivan et al., 2014; Pet et al., 2018) and speech and language development problems, not only because of adaptation to a new language but also anatomically (Scherer et al., 2018). However, these higher revision rates and the higher risk of velopharyngeal insufficiency is not uniformly reported in the literature (Goldstein et al., 2014).

### Quality of Life in Cleft lip and/or Palate

The problems with eating, drinking, speaking, hearing and dentition that all children with CL/P may experience are known to influence psychosocial well-being of the child. Although demographics and treatment of adopted children with cleft lip and/or palate has been studied before, few studies specifically address quality of life. The aim of the current study was to compare quality of life of internationally adopted children with CL/P to a propensity score matched sample of non-adopted children with CL/P.

## Methods

A cross-sectional study involving children treated by the CL/P treatment teams of our region was performed. The study was approved by the Institutional Review Boards of the Medical Center Leeuwarden and the University Medical Center Groningen, the Netherlands. All parents of participants provided written consent prior to participation.

### Participant Selection

All children with CL/P treated by the multidisciplinary CL/P treatment teams of the Medical Center Leeuwarden and University Medical Center Groningen aged 8 years old or younger were initially eligible for participation. Children with syndromal types of cleft lip and/or palate and parents who did not speak Dutch were excluded from participation. Since we know the vast majority of adopted children at our institution are originally from China this was the focus of this study, children with other countries of origin were excluded. The control group of patients were all white children born in the Netherlands.

### Data Collection

Eligible participants received a letter at home inviting them to participate. A letter with a reminder was sent after 4 weeks in case of non-response. In case of participation, a questionnaire was sent out consisting of some demographic questions about the child's gender, age and country of origin, the highest level of education of the parent filling out the questionnaire. Additionally, patient files were searched for clinical data such as type of cleft, treatment stage and other relevant health data.

### CleftChild-8

The CleftChild-8 (CC-8) is a parent proxy instrument for CL/P-related quality of life and patient satisfaction in children with CL/P up to eight years old (Busschers, 2016). The questionnaire contains 43 Likert scale type questions that can be divided in four domain scores and 13 subdomain scores (Table 1). Questions are scored from 1 to 5 with several different answer options depending on the type of question (eg rating how satisfied they are with something, or how often something is experienced). The scores of the individual questions are summed to create a domain score with higher scores meaning better quality of life or patient satisfaction. In case of a missing value, (sub)domain scores are calculated based on the available scores within the (sub)domain of interest.

**Table 1.** CleftChild-8 Domain and Subdomain Scores.

1. Satisfaction with cleft lip and/or palate team functioning
1.1 Cleft team guidance
1.2 Peer contact group
1.3 Cleft team website
1.4 Importance of regional treatment
2. Satisfaction with (operative) treatment
2.1 Peri-operative care
2.2 Post-operative results
3. Psychological wellbeing and social relationship
3.1 Social functioning
3.2 Acceptance by siblings
3.3 Acceptance by family/friends
3.4 Satisfaction with acceptance by family/friends
3.5 Influence of cleft lip and/or palate on life in general
4. Daily functioning
4.1 Communication problems
4.2 Additional health problems due to cleft lip and/or palate

The questionnaire was developed in 2016 and shown to have adequate psychometric properties (Busschers, 2016). Internal consistency of the questionnaire (sub)domains was shown to be adequate with Cronbach's α in the range of 0.667 to 0.955. Only two subdomains had an Cronbach's α below the 0.7 treshold: ‘importance of regional treatment’ (0.667) and ‘additional health problems due to cleft lip and/or palate’ (0.699). Validity of the CC-8 was studied by comparing CC-8 scores to relevant scores of the TACQOL questionnaire (health-related quality of life questionnaire validated for children). Eight hypotheses were tested, and four accepted. The CC-8 scores showed good validity for the questions concerning speech, difficulty eating, difficulty sleeping, and having feelings of anger and sadness. Lastly, test-retest reliability was studied with a two week interval and found to be acceptable for all (sub)scales (Pearson correlation >0.737).

### Statistical Analysis

Descriptive statistics were presented by frequencies and percentages, means and standard deviations (SD) and medians and interquartile ranges (IQR) as appropriate. Differences between the adopted and non-adopted children were determined by Fisher's exact test for nominal data, independent T-test for normally distributed continuous data and Mann Whitney U test for non-normally distributed continuous data and ordinal data.

Since we expected the two groups to be significantly different on various characteristics, we used propensity score matching to balance the groups. A propensity score can be considered as an a priori probability of a participant to be included in the experimental group, in this case the adopted children group, given a set of characteristics. By matching the participants based on the propensity score, we matched adopted children with controls having the same likelihood to be part of the adopted children group. The propensity score was calculated using a logistic regression model based on the variables sex, age, type of cleft, level of education of the parents and whether or not palatal surgery had been performed. Palatal surgery was added to our model in an attempt to balance the groups to some degree on treatment stage. Several types of propensity score matching were tested and compared. Propensity score matching using the ‘nearest neighbor’ algorithm with a 1 to 1 ratio resulted in the smallest standardized mean differences in propensity scores and was therefore performed. Patient characteristics were compared between the two matched groups to determine if the matching procedure was successful. Lastly, CC-8 scores were compared between the two matched groups. Comparison in the matched cohort was done using the McNemar's test for nominal dichotomous data, the marginal homogeneity test for nominal data with more than two categories, paired sample T-test for normally distributed continuous data and Wilcoxon signed-rank test for non-normally distributed continuous data and ordinal data. Effect size (r) was calculated according to the formula ‘r = Z-value / √N’ and interpreted using the Cohen (1988) criteria of.1 = small effect,.3 = medium effect and.5 = large effect.

Propensity score matching was performed using R software (R Foundation for Statistical Computing version 3.6.3 (Vienna, Austria)), all other analyses were performed using the Statistical Package for the Social Sciences (SPSS) version 23 (NY, USA) with a *P*-value of ≤.05 as the threshold for statistical significance.

## Results

**Figure 1.**
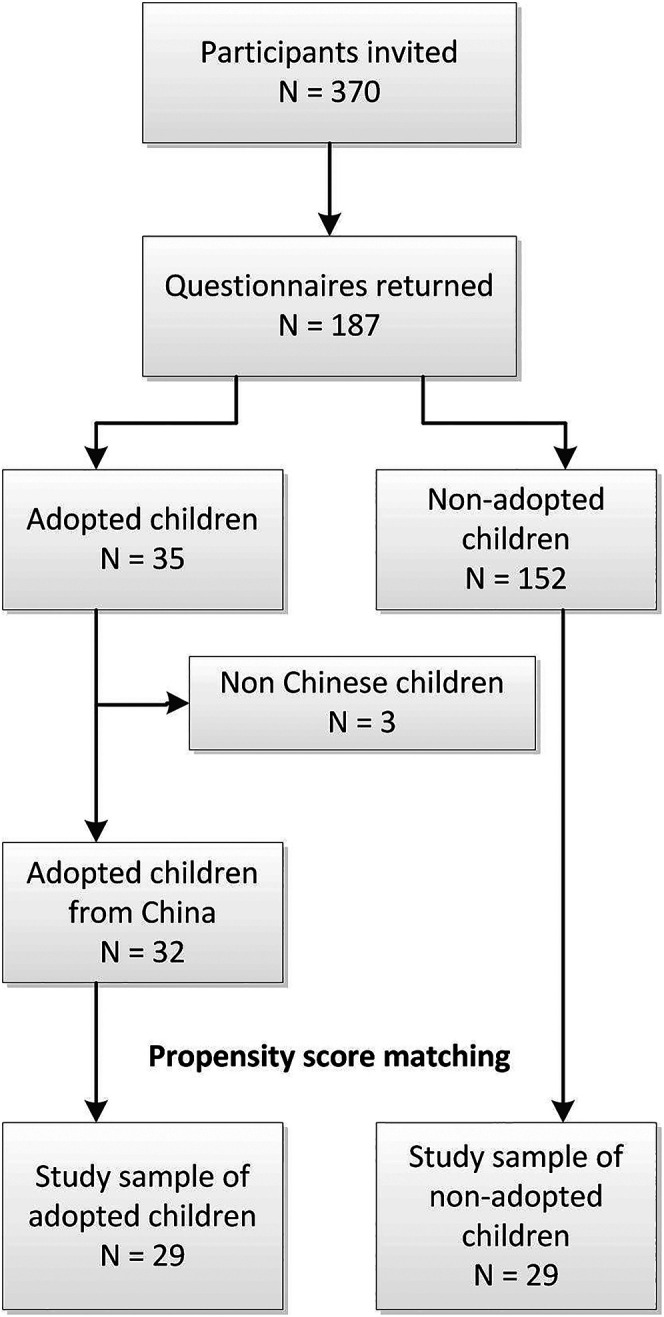
Flowchart of the participant selection and propensity score matching process.

Of the 370 eligible children identified, the parents of 187 (50.5%) children returned the questionnaires. Thirty-five (18.7%) children were adopted, most (n = 32 (91.4%)) from China. One child from Taiwan, one child from Bulgaria and one child with an unknown country of origin, were excluded from the analyses. The 152 non-adopted children were locally born in a largely white region (>97.5%) (University College London, 2001); the control group did not include any locally born children of Asian descent. In order to make comparable groups of adopted and non-adopted children, a propensity score matching procedure was performed. The propensity score matching procedure resulted in 29 pairs of participants (Figure 1). Before matching, adopted children had different cleft phenotypes and a trend toward more boys and a higher level of education of the parent amongst the adopted children. After matching, participant characteristics and parental education level between the adopted and non-adopted children were comparable (Table 2).

**Table 2.** Participant Characteristics Before and After Propensity Score Matching.

	Before matching			After matching		
	Adopted (n = 35)	Not adopted (n = 152)	*P*-value	Adopted (n = 29)	Not adopted (n = 29)	*P*-value
Male gender (n (%))	27 (77.1)	93 (61.2)	0.082^a^	22 (75.9)	21 (72.4)	1.000^c^
Age, in years (median (IQR))	5.8 (4.6; 7.5)	5.8 (2.9; 7.5)	0.405^b^	5.8 (4.7; 7.4)	6.8 (4.6; 7.8)	0.654^d^
Cleft phenotype (n (%))			**<0.001** ^a^			1.000^e^
Complete bilateral cleft lip (and palate)	14 (40.0)	16 (10.5)		13 (44.8)	13 (44.8)	
Incomplete bilateral cleft lip (and palate)	1 (2.9)	7 (4.6)		—	—	
Complete unilateral cleft lip (and palate)	18 (51.4)	32 (21.1)		14 (48.3)	14 (48.3)	
Incomplete unilateral cleft lip (and palate)	1 (2.9)	61 (40.1)		1 (3.4)	1 (3.4)	
Isolated cleft palate	1 (2.9)	36 (23.7)		1 (3.4)	1 (3.4)	
Palatal surgery performed (n (%))	18 (51.4)	69 (45.4)	0.575^a^	17 (58.6)	19 (65.5)	0.500^c^
Level of education parents (n (%))			0.093^a^			0.656^e^
Primary or secondary education	4 (11.4)	21 (13.8)		3 (10.3)	4 (13.8)	
Vocational education	9 (25.7)	66 (43.4)		8 (27.6)	9 (31.0)	
Higher education	22 (62.9)	65 (42.8)		18 (62.1)	16 (55.2)	

Abbreviations: IQR – interquartile range; n – number.

^a^ Result of Fisher’s Exact test.

^b^ Result of Mann Whitney U test.

^c^ Result of McNemar’s test.

^d^ Result of Wilcoxon Signed Ranks test.

^e^ Result of Marginal Homogeneity test.

Depending on the type of cleft and age of the child, the children were in differing stages of their treatment. Nine complications (five palatal fistulae, three cases of wound dehiscence and one infection) in seven (24.1%) adopted children were observed compared to nine complications (three palatal fistulae, three cases of wound dehiscence and three cases of remaining nasal speech after pharyngoplasty) in eight (27.6%) non-adopted children (*Χ*^2^ = 0.090, *P* = 1.00). Re-operation because of the complication was seldom needed (n = 5 (17.2%) for the non-adopted children versus n = 3 (10.3%) for the adopted children (*Χ*^2^ = 0.580, *P* = .688)). We looked in detail into the age at palatal closure in our sample, since it is reported to differ in literature and may have consequences for the further development of the child. Median (IQR) age at the time of palatal closure was 0.9 (0.9; 1.1) years for the non-adopted children versus 2.4 (2.0; 3.1) years for the adopted children with CL/P (*P* = 0.007). Hearing health was comparable between both groups with 5 (17.2%) adopted children needing PE tube placement versus 2 (6.9%) non-adopted children (*Χ*^2^ = 1.462, *P* = .453). Speech and language therapy was reported for 10 (34.5%) adopted children versus 13 (44.8%) non-adopted children (*Χ*^2^ = 0.648, *P* = 0.648).

Quality of life of the adopted and non-adopted children with CL/P was compared by use of the CC-8. Median (IQR) CC-8 scores for adopted children were lower compared to the non-adopted children on 12 of the 17 (70.6%) (sub)domain scores (Table 3), meaning lower quality of life for adopted children versus non-adopted children. A significant difference was seen for the domain ‘satisfaction with (operative) treatment’ (median (IQR) scores of 31 (24; 34) versus 34.5 (31.25; 38), *P* = .011, *r* = 0.465) including questions regarding the operation results and peri-operative care. The subdomain scores for ‘postoperative results’ (median (IQR) scores of 11.5 (4.25; 13) versus 13 (12; 14), *P* = .004, *r* = 0.524) were also significantly different. The subdomains ‘acceptance by siblings’ (median (IQR) scores of 12 (8.5; 14) versus 14 (12.5; 15), *P* = .014, *r* = 0.448) and ‘acceptance by family/friends’ (median (IQR) scores of 10 (7; 10) versus 10 (9.5; 10), *P* = .049, *r* = 0.364) were significantly different meaning that parents of adopted children reported lower social acceptance scores by siblings and relatives/friends compared to the parents of non-adopted children (Table 3). As we report on 17 comparisons between scores of adopted versus non-adopted children, a multiple testing problem may arise. In our study this would result in zero (sub)domains differing at the Bonferroni correct p-value of 0.003 partly due to a small sample size.

**Table 3.** Median (Interquartile Range) CleftChild-8 Scores of Propensity Score Matched Adopted Versus not Adopted Children with Cleft lip and/or Palate.

CleftChild-8 (sub)domain	Adopted children (n = 29)	Non-adopted children (n = 29)	*P*-value^a^	Z-value	Effect size r^b^
1. Satisfaction with cleft lip and/or palate team functioning	45 (38.5; 52.5)	48 (43; 51)	0.286	1.082	0.201
1.1 Cleft team guidance	29 (27.5; 32)	31 (28; 34.5)	0.093	1.688	0.313
1.2 Peer contact group	3 (0; 6.5)	3 (0; 6.5)	0.993	0.016	0.003
1.3 Cleft team website	6 (3; 8)	7 (2.5; 8)	0.895	0.144	0.027
1.4 Importance of regional treatment	9 (8; 10)	9 (8; 10)	1.000	0.015	0.003
2. Satisfaction with (operative) treatment	31 (24; 34)	34.5 (31.25; 38)	**0.011**	2.505	0.465
2.1 Peri-operative care	20 (18; 21)	21 (20; 25)	0.059	1.887	0.350
2.2 Post-operative results	11.5 (4.25; 13)	13 (12; 14)	**0.004**	2.820	0.524
3. Psychological wellbeing and social relationship	62 (56.5; 66)	66 (60; 67)	0.413	0.833	0.155
3.1 Social functioning	26 (21; 30)	26 (21.5; 28.5)	0.996	0.011	0.002
3.3 Acceptance by siblings	12 (8.5; 14)	14 (12.5; 15)	**0.014**	2.412	0.448
3.5 Acceptance by family/friends	10 (7; 10)	10 (9.5; 10)	**0.049**	1.956	0.364
3.2 Satisfaction with acceptance by family/friends	10 (8; 10)	10 (9; 10)	0.155	1.509	0.280
3.4 Influence of cleft lip and/or palate on life in general	13 (11; 14)	14 (12; 15)	0.166	1.399	0.260
4. Daily functioning	21 (17; 26)	23 (22; 26.5)	0.075	1.789	0.332
4.1 Communication problems	14 (11.5; 17.5)	16 (13.5; 17)	0.120	1.566	0.291
4.2 Additional health problems due to cleft lip and/or palate	7 (6; 9)	8 (6.5; 9)	0.214	1.267	0.235

Bonferroni correction for multiple testing of 17 statistical tests would result in a significance level op *P* <.003. None of the 4 differences found would be considered significant at this *P*-value.

a Result of Wilcoxon Signed Ranks test.

b Calculated as ‘Z-value/√N’.

CC-8 scores divided by cleft phenotype are shown in supplementary table 1. A statistical comparison of quality of life of different cleft phenotypes was outside the scope of this manuscript, and not performed due to small group sizes (with two groups of n = 1), but no large differences in CC-8 scores between different cleft phenotypes were seen (supplementary table 1).

## Discussion

In the current study, quality of life of adopted children with CL/P was compared to a propensity score matched sample of non-adopted children with CL/P. Quality of life of adopted children was found to be significantly lower on several (sub)domain scales.

Parents of adopted children are significantly less satisfied with the result of various surgical procedures. As noted, adopted children with CL/P are usually a few years older at the time of first presentation. Hence, some of the surgical procedures are performed later than we would prefer. For example, palatal closure in our sample was performed at a median age of 0.9 years in non-adopted children, while adopted children were a year and a half older. Not operating at the ideal time may lead to more functional problems and more secondary corrections later in life. Our study shows that this is not the opinion of the surgeon, but also the opinion of the parents of adopted children. This information could be used in counseling and expectation management of families of adopted children and addressing possible underlying feelings of nonsatisfaction with parents early.

Additionally, the adopted children scored lower on acceptance by siblings and acceptance by family/friends. Although we cannot fully explain these findings we have several hypotheses for these lower scores. One hypothesis is that in the largely white population of our region the internationally adopted children may stand out more in their local community compared to the non-adopted children. Latent racism could be a contributing factor, as reports on discrimination of people of Chinese descent increase since the current COVID19 pandemic (Broekroelofs, 2020). However, in general children adopted from China are reported to perform well in Dutch society with higher levels of education compared to native Dutch children (Centraal Bureau voor Statistiek, 2015). Another explanation may be that the lower scores of internationally adopted children reflect some degree of relationship problems within the family unit, as these types of problems are known to be more prevalent in case of special needs adoptions compared to ‘no special needs’ adoptions (Child Welfare Information Gateway, 2012).

Adopted children with CL/P have been topic of study previously, however, quality of life of these children has not. By performing propensity score matching, we have compared quality of life of these adopted children to non-adopted children with CL/P while correcting for other potentially influencing factors such as the type and severity of the cleft and the level of education of the parents. We were able to include 29 adopted children in our analysis, which is in line with other studies, but it remains a small number of study participants. Future studies in more CL/P treatment centers would increase the number of study participants and add strength to the study findings.

Due to the cross-sectional design of our study we could only examine a snapshot in time. A future longitudinal study would provide insight in the development of quality of life as children grow older. International adoption is known to be associated with a lower general health, malnutrition and development delays (Miller et al., 1995; Miller and Hendrie, 2000; Mason and Narad, 2005; Cohen et al., 2008; Park et al., 2011) which could influence treatment (outcomes) and quality of life. Swanson et al. (2014) observed improvements in all growth percentiles after adoption. Perhaps differences in quality of life between internationally adopted and non-adopted children with CL/P dissolve with time along with the improvement in general health.

In the current study, we tried to correct for potential confounding variables such as hearing health, supportive non-operative treatments and complications. However, medical files were not always complete or fully available and it is difficult to correlate findings from the medical file to the CC-8 scores since they are not from the same point in time. Future studies should be performed to examine the relationship between specific health problems, such as velopharyngeal insufficiency, middle ear problems, and problems with dentition, and quality of life. Especially set in a longitudinal study design, this could provide information regarding causation of quality of life. Additionally, our study does not include information regarding the adoption process or pre-adoption data, which makes it difficult to interpret results for individual patients. Future studies should investigate if difficulties around the adoption process or pre-adoption quality of care influences cleft-specific quality of life and satisfaction with care after adoption.

Our use of the CC-8 limited our study to children younger than eight years old. Additionally, the CC-8 is a patient proxy instrument. Although a much used solution in very young children, a parent proxy instrument remains the parents view of the patients’ quality of life. A future study should be performed in older children, perhaps using an instrument such as the CLEFT-Q which is suitable for children and young adults aged 8–29 years (Klassen et al., 2018), to see if differences in quality of life remain or resolve over time and to assess the influence of our use of a parent proxy instrument instead of a self-reported questionnaire. Also, two of the four significantly differing CC-8 scales were within the ‘satisfaction with (operative) treatment’ domain. The questions of the CC-8 are formulated in such a way that, especially in a cross-sectional setting, it is difficult to disentangle if parent report on only the results of the last operation or the operative results in general. A longitudinal study would be more suitable to examine satisfaction with a single procedure. However, the fact remains that parents of adopted children are less satisfied with the results at the point of measurement, whether those are pre-adoption procedures or not.

One of the more difficult possibly confounding variables in our study was age at the time of palatal closure. Palatal surgery was incorporated into our propensity score matching model as part of correcting for this confounding variable. However, age at the time of palatal surgery could not be corrected for since this was so distinctly different for the adopted children versus the non-adopted children. A study including children adopted at such a young age that palatal closure can be performed within the standard timeframe would help to disentangle the effect of adoption versus the effect of older age at the time of palatal closure.

## Conclusions

Our study demonstrates that adopted children with CL/P experience lower quality of life compared to non-adopted children with CL/P, with a significantly lower quality of life on several domains compared to non-adopted children. Members of CL/P teams should be aware of this and perhaps offer adopted children additional guidance and counseling. Although adopted children do not form the majority of patients presented to a CL/P treatment team, a treatment and guidance program tailored to the specific needs of adopted children is recommended.

## Supplemental Material

sj-docx-1-cpc-10.1177_10556656211050795 - Supplemental material for Quality of Life of Adopted Chinese Versus Nonadopted Dutch Children with Cleft Lip and/or Palate: A Propensity Score Matched Analysis Click here for additional data file. Supplemental material, sj-docx-1-cpc-10.1177_10556656211050795 for Quality of Life of Adopted Chinese Versus Nonadopted Dutch Children with Cleft Lip and/or Palate: A Propensity Score Matched Analysis by Martinus M. van Veen, Bente A. van den Berge and Chantal M. Mouës-Vink in The Cleft Palate-Craniofacial Journal
