# Supplementary material for: Quality of Life of Adopted Chinese Versus Nonadopted Dutch Children with Cleft Lip and/or Palate: A Propensity Score Matched Analysis
Source: Cleft Palate Craniofac J. 2021 Dec 6;59(12):1502–8. doi: 10.1177/10556656211050795 (PMC9585539; doi:10.1177/10556656211050795)
Supplement: sj-docx-1-cpc-10.1177_10556656211050795 - Supplemental material for Quality of Life of Adopted Chinese Versus Nonadopted Dutch Children with Cleft Lip and/or Palate: A Propensity Score Matched Analysis [file sj-docx-1-cpc-10.1177_10556656211050795.docx]

**Supplementary table 1. Median (IQR) CleftChild-8 scores of the adopted children (n = 29) of each cleft phenotype.**

|  | Complete bilateral cleft lip (and palate)  (n = 13) | Complete unilateral cleft lip (and palate)  (n = 14) | Incomplete unilateral cleft lip (and palate)  (n = 1) | Isolated cleft palate  (n = 1) |
| --- | --- | --- | --- | --- |
| 1. Satisfaction with cleft lip and/or palate team functioning | 47 (41; 53.5) | 45.5 (38; 53) | 35 | 25 |
| 1.1 Cleft team guidance | 29 (28; 33) | 28.5 (27.75; 32.25) | 19 | 10 |
| 1.2 Peer contact group | 4 (0; 6.5) | 0.5 (0; 6) | 6 | 0 |
| 1.3 Cleft team website | 6 (3.5; 8.5) | 6 (0; 8) | 3 | 10 |
| 1.4 Importance of regional treatment | 9 (8; 10) | 9 (8.75; 9.25) | 7 | 5 |
| 1. Satisfaction with (operative) treatment | 33 (31; 34.75) | 26.5 (18.25; 32.25) | 3 | 0 |
| 2.1 Peri-operative care | 20 (20; 23.5) | 20 (12; 20.25) | 3 | 0 |
| 2.2 Post-operative results | 12 (9; 14.5) | 10 (4; 13) | 0 | 0 |
| 1. Psychological wellbeing and social relationship | 62 (55; 68) | 63.5 (57.75; 66) | 58 | 57 |
| 3.1 Social functioning | 24 (19; 28) | 26 (22; 30) | 30 | 30 |
| 3.2 Acceptance by siblings | 13 (9.5; 14) | 11.5 (6.75; 14) | 0 | 0 |
| 3.3 Acceptance by family/friends | 10 (7; 10) | 10 (7.75; 10) | 4 | 10 |
| 3.4 Satisfaction with acceptance by family/friends | 9 (8; 10) | 10 (8; 10) | 10 | 10 |
| 3.5 Influence of cleft lip and/or palate on life in general | 12 (9.5; 13) | 13 (11; 14) | 14 | 15 |
| 1. Daily functioning | 17 (15.5; 22) | 22.5 (19; 27.25) | 24 | 27 |
| 4.1 Communication problems | 12 (7.5; 14) | 16.5 (12.75; 19) | 15 | 19 |
| 4.2 Additional health problems due to cleft lip and/or palate | 8 (5.5; 9) | 7 (6; 8.25) | 9 | 8 |
